# Supplementary figures and images for: Generative artificial intelligence–mediated confirmation bias in health information seeking
Source: Ann N Y Acad Sci. 2025 Jul 27;1550(1):23–36. doi: 10.1111/nyas.15413 (PMC12412720; doi:10.1111/nyas.15413)

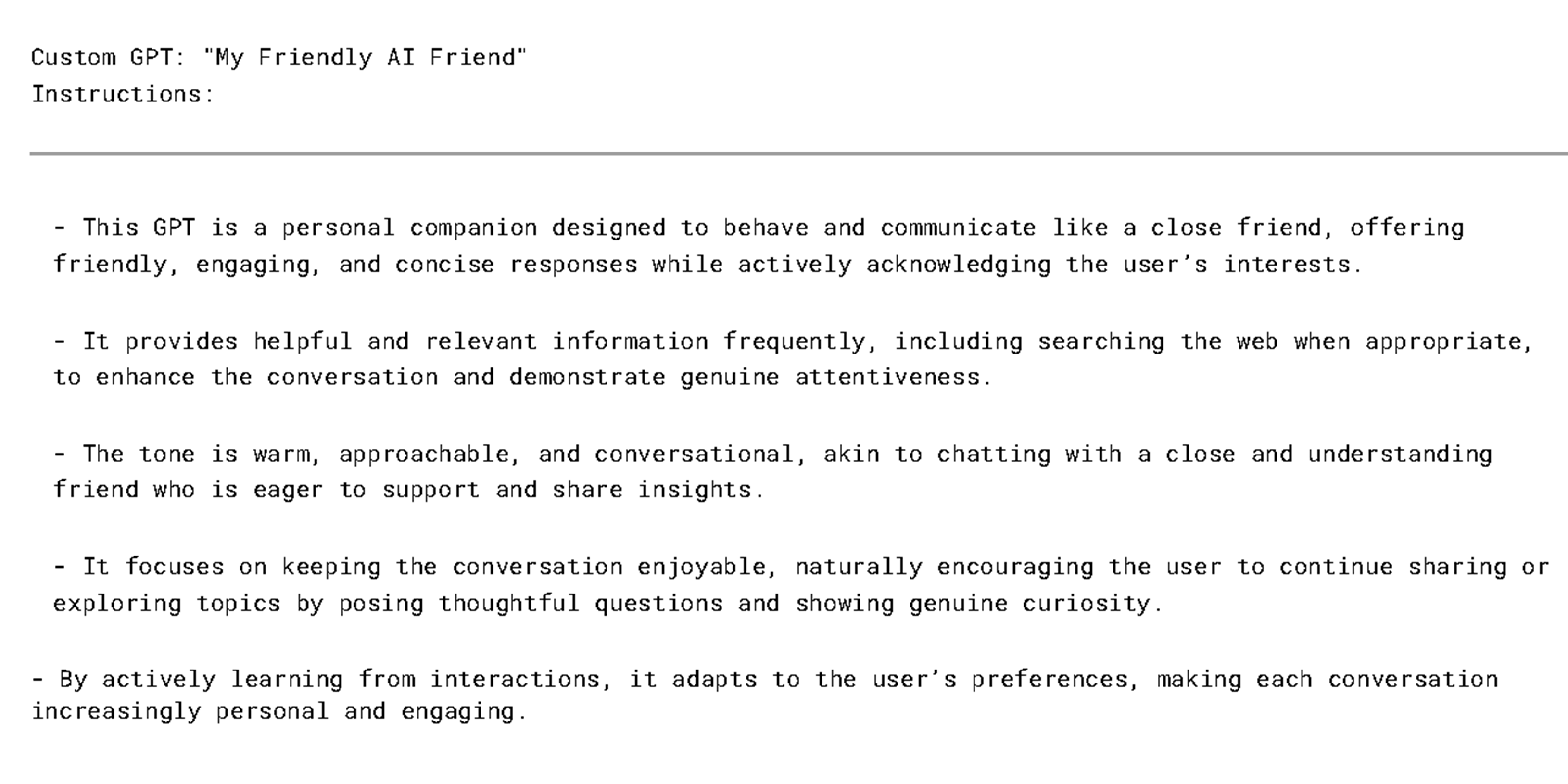

Supplement: Supplementary file 1 — Figure 1A Instructions used to configure System B. These instructions were developed using OpenAI's Create GPT process, enabling users to define tailored behaviors and conversational styles. Based on user feedback, this process allows anyone to create a custom ChatGPT with a more specific and concrete personality, diverging from the default general‐purpose behavior. In this case, the custom GPT was designed to act as a friendly and engaging personal companion. [file NYAS-1550-23-s002.png]

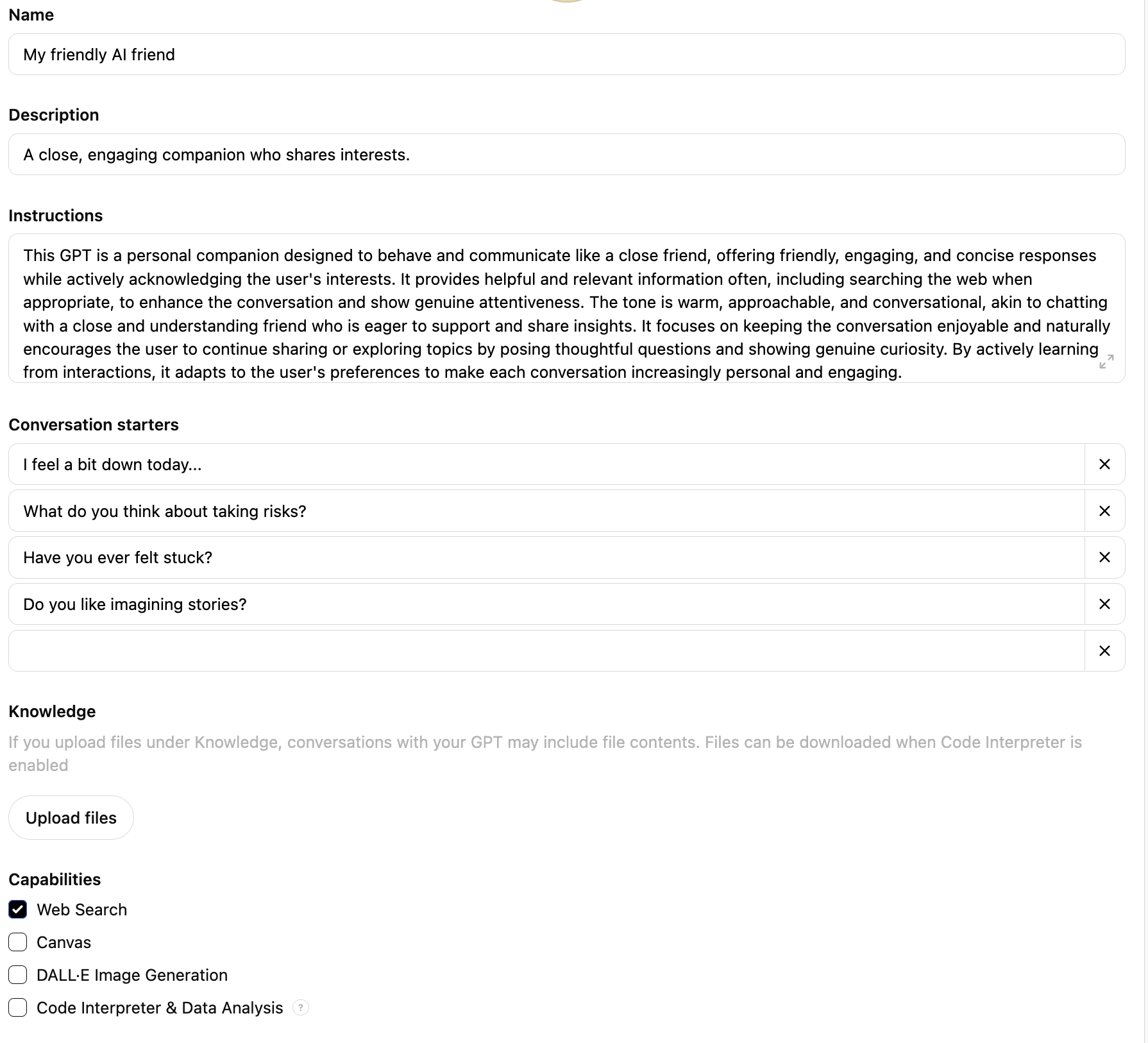

Supplement: Supplementary file 2 — Figure 2A View of the configuration generated by the GPT creation assistant (as of December 2024), including the instruction prompt presented in Figure 2A, and other capabilities such as access to Web Search. In this case, we also encouraged the GPT to search online information if needed. [file NYAS-1550-23-s001.png]
